# The Best Time for EEG Recording in Febrile Seizure

**Published:** 2014

**Authors:** Parvaneh KARIMZADEH, Alireza REZAYI, Mansoureh TOGHA, Farzad AHMADABADI, Hojjat DERAKHSHANFAR, Eznollah AZARGASHB, Fatemeh KHODAEI

**Affiliations:** 1 Pediatric Neurology Research Center, Shahid Beheshti University of Medical Sciences (SBMU), Tehran, Iran; 2 Pediatric Neurology Excellence Center, Pediatric Neurology Department, Mofid Children Hospital, Faculty of Medicine, Shahid Beheshti University of Medical Sciences (SBMU), Tehran, Iran; 3 Neurology Department, Sina Hospital, Tehran University of Medical Science, Tehran, Iran; 4 Pediatric Emergency Department, Mofid Children’s Hospital, Faculty of Medicine, Shahid Beheshti University of Medical Sciences, Tehran, Iran; 5 Social Medicine Department, Faculty of Medicine, Shahid Beheshti University of Medical Sciences, Tehran, Iran; 6 Department of Midwifery, School of Nursing & Midwifery, Isfahan University of Medical Sciences, Esfahan, Iran

**Keywords:** Simple, Complex, Febrile seizures, Electroencephalography, Epileptiform discharges

## Abstract

**Objective:**

Some studies suggest that detection of epileptic discharge is unusual during the first postictal week of febrile seizure and others believe that EEGs carried out on the day of the seizure are abnormal in as many as 88% of the patients. In this study, we intend to compare early and late EEG abnormalities in febrile seizure.

**Materials & Methods:**

EEG was recorded during daytime sleep, 24-48 hours (early EEG) and 2 weeks (late EEG) after the seizure in 36 children with febrile seizure (FS), aged between 3 months and 6 years. EEGs that showed generalized or focal spikes, sharp, spike wave complex, and slowing were considered as abnormal EEG. Abnormalities of the first EEG were compared with those of second EEG.

**Results:**

The most common abnormal epileptiform discharges recorded in the early EEG were slow waves (27.6%) and sharp waves in late EEG (36%). Distribution of abnormalities in early and late EEG showed no significant statistical difference.

**Conclusion:**

The early and late EEG recording had the same results in patient with febrile seizure.

## Introduction

Febrile seizures (FS) are common disorder in 3-month- to 6-year-old children, with an incidence varying between 2% and 5% (1). It is associated with fever, without evidence of intracranial infection or a definite cause (2). These seizures are classified as simple or complex (3). Simple FS is a generalized tonic-clonic seizure of less than 10 minutes duration that does not recur within the subsequent 24 hours. Complex FS is defined in case one or more of the following features are present: a focal onset or focal feature during the seizure, prolonged duration of more than 15 minutes, and recurrence during 24 hours (4-7).

In a case of patient, an Electroencephalography (EEG) recording is not usually indicated for evaluation either in the hospitalized or outpatient settings (8). EEGs are most helpful if there is any doubt about whether FS has really occurred, because EEGs done on the day of seizure are abnormal in as may as 88% of patients (9).

The effectiveness of EEG for prediction of subsequent epilepsy or recurrent FS has not been sufficiently evaluated, and the role of EEG in the work-up of FS is still controversial. Many pediatric neurologists continue to request EEG after an initial FS (10).

The timing of the EEG has to be considered as generally. Detection of epileptic discharge is unusual in the first postictal week and it is also difficult before the age of 3 years (11-13).

In this study, we evaluated the rate of EEG abnormalities in children with FSs, shortly after the FS and 2 weeks later.

## Materials & Methods

This study was performed on 36 children aged between 3 months and 6 years with the diagnosis of FS. FS was defined as a convulsion associated with fever, without a history or evidence of preexisting neurological abnormality, metabolic disorder, or intracranial infection. All children were managed according to a routine protocol including fever reduction or seizure control and other investigations. Then, we recorded the demographic information, history, physical examination, and type of seizure in the patient’s questionnaire. In this study, EEG was recorded in two phases: shortly after the FS and 2 weeks later. So, first and second EEGs were parts of a systematic protocol. 

EEG was recorded during daytime sleep, 24-48 hours (early EEG), and 2 weeks (late EEG) after the seizure with the administration of oral chloral hydrate (50-75 mg/kg). We used “Wega 10” EEG equipment, and in all children, recording was performed during sleep for at least 20 minute.

 Children with previous nonfebrile seizure, patients with evidence of intracranial infection, patients with electrolyte imbalance, or those who were not referred for second EEG were excluded. 

EEGs were interpreted by a pediatric neurologist, who was unaware of the patient’s previous EEG. EEGs that showed generalized or focal spikes, sharp, spike wave discharge, and slowing (including high voltage slow wave) were considered as abnormal. Patients’ history information and EEG data were collected in questionnaires, and then statistical analysis was performed using SPSS software.

## Results

During the study time, 58 children with the diagnosis of FS were admitted to the emergency unit of Mofid Children’s Hospital. A total of 36 patients (24 boys and 12 girls) met the inclusion criteria. The mean age of the patients was 28 months (range, 4-72 months). 

**Graph 1:**
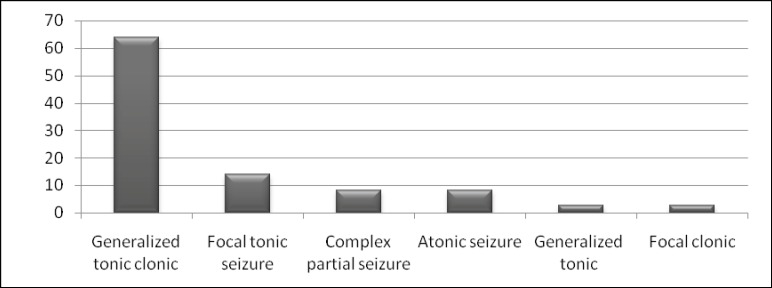
Frequency of different seizure types among patients

The most common seizure type was generalized tonicclonic seizure (Graph 1).

Twenty-three (63.9%) patients were classified as complex FS and thirteen (36.1%) as simple FS. Early EEG abnormality was reported in 29 (80.6%) patients and late EEG showed abnormality in 25 patients (69.4%). 

**Fig 1:**
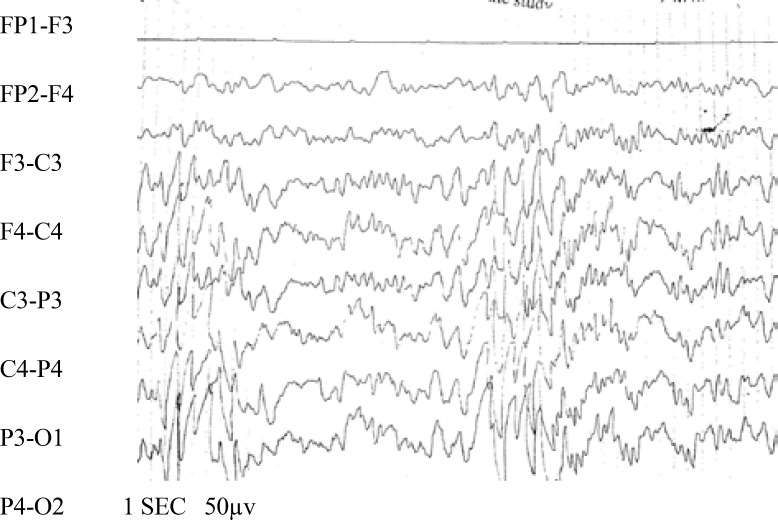
Early EEG of a 3-year-old girl with a complex FS (spike wave and sharp wave discharge)

**Fig 2:**
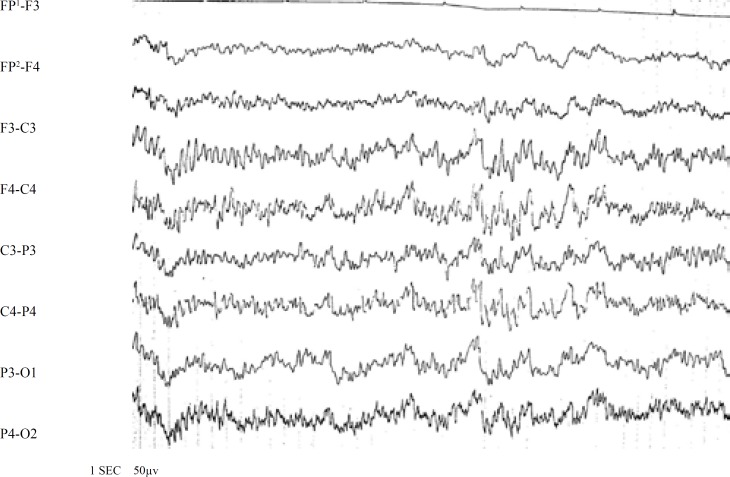
Late EEG of a 15-month-old girl with complex FS (spike in the rolandic region)

**Fig 3:**
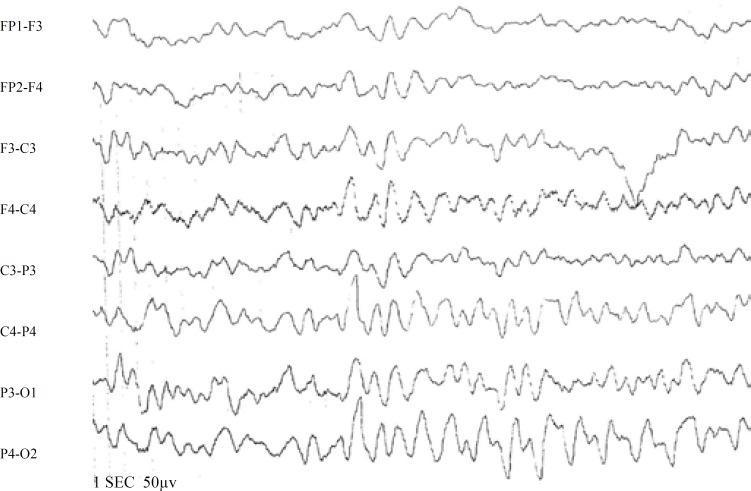
Early EEG of a 6-year-old boy with simple FS (delta activity in right side)

Statistical analysis of comparison between early and late EEG abnormalities in patients with FS did not show significant difference between the two groups. The most common abnormal discharges recorded in the early EEGs were slow waves (31%) and sharp waves (27.6%). Sharp waves were recorded as the most common waves (36%), and a combination of spike waves and sharp waves (28%) were the second most common in late EEGs. On the other hand, these EEGs showed spike waves in one montage and sharp waves in another montage (Table 1 and Figure 1-3). Of the 13 patients with complex FS whose late EEGs were abnormal, 3 patients (8.3%) had a normal early EEG, and 10 patients (27.7%) had an abnormal early EEG. 

Table 2 shows the localization of paroxysmal discharge in early and late EEG.

In patients with complex FS aged less than 3 years, early EEG abnormality was detected in two-third (7 of 10) of the patients; however, this abnormality was detected in all patients more than 3 years of age. Seven children (19.4%) had a history of FS and 5 children (13.9%) had a positive family history of FS. In all the patients with a positive family history of FS, early EEG abnormality was seen.

## Discussion

A Review of the published literature suggests that 2-86% of the EEGs are abnormal after FS (14). This variation may be due to authors’ different methods, different definition of EEG abnormality, age, timing of the EEG, and the effect of viral infection on EEG (15). 

The present study shows that abnormal EEG findings are not influenced by the time of EEG recording. The incidence of early EEG abnormality in our study was 80.6% in comparison with 69.4% abnormality in late EEG. The timing of the EEG in other literature has been shown to be an important variable in determining the presence of epileptiform abnormality. In Lennox-Buchthal’s study, epileptiform EEG abnormality was rare within the 7-day period after FS (16).

In a few studies, slowing was found to be more prevalent in the early postictal period (14,17-19). In our study, the most common abnormal discharge was slow wave (31%) in the early postictal EEG and sharp wave was the second most common wave (27.6%). In a sequential study of patients with uncomplicated FSs, 33% had prominent delta activity diffusely or over the posterior head region. This activity resolved within 2 weeks and had no prognostic significance (17). In the present study, delta activity in early EEG was recorded in nine patients (31%), of which 24% were diffuse and 7% were in the bioccipital region. These abnormalities resolved after 2 weeks (late EEG) in 4 patients (13.7%), however persistent delta activity was seen in 5 patients (17.3%). In another study, EEG abnormality was 22.5% within 6 days after complex FS, and our results showed that early EEG abnormality in complex FS was 27.7%. In a study by Maytal et al., the incidence of paroxysmal abnormality on EEG in neurologically normal children within a week of complex FS was 0% with a calculated true rate of abnormality of 8.6% or less (14).

In a study by Joshi et al., the results showed that children with complex FS are approximately 3.5 times more likely to show an abnormal EEG in 2 days after ictus compared to children with complex FS, when the EEG was done beyond the 7-day postictal period (15). 

The results of this study were similar to the results of our study that showed 27.7% of children with complex FS had an abnormal early EEG, and 69.2% of these patients had abnormalities in late EEG. 

EEG findings have changed with the age of the child at the time of the first complex FS. The study of Aicardi and Chevrie showed that detection of an epileptiform pattern is infrequently seen in children with FS aged less than 3 years (12). The result of our analysis supports a similar conclusion.

This study showed that early epileptiform EEG abnormality was seen in children older than 3 years of age and in two-third of children younger than 3 years of age. The results of our study showed that 13.9% of the children had a positive family history of FS. In all these children, early epileptiform EEG abnormality was detected.

This finding does not support Joshi et al.’s study, which showed a lower proportion of epileptiform abnormality in patients with a positive family history of FS (15). 

**In conclusion, **the literature shows that age, an abnormal neurological examination, a positive family history of FS, and a 7-day postictal timing of the recording significantly influence the likelihood of abnormal findings on the EEG; however, our study was focused on the timing of the EEG recording after seizure and concluded no association between abnormal epileptiform discharge and early - late EEG recording. 

**Table 1:** Frequency of Abnormal Discharges In Abnormal Early And Late EEGs According To The Wave Type

**Wave**	**Early abnormal EEG (%)**	**Late abnormal EEG (%)**
**High voltage slow wave**	31	20
**Sharp**	27.6	36
**Sharp+spike** *	13.8	28
**Spike**	10.3	4
**Spike wave discharge**	3.4	0
**Spike wave discharge+ sharp wave** €	3.4	4
**Other** #	10.5	8
**Total**	100	100

* This EEGs showed spike waves in one montage and sharp waves in another montage.

€ This EEGs showed spike waves discharge in one montage and sharp waves in another montage

# Low voltage slow wave, sharp wave+high voltage slow wave, spike wave discharge+ high voltage slow wave, spike+high voltage slow wave

**Table 2:** Localization of Abnormal Discharge In Early And Late EEGs

**EEG Localization**	**Early EEGs**	**Late EEGs**
Generalized	22 (61.2%)	16 (44.5%)
Rolandic	2 (5.5%)	3 (8.3%)
Occipital	1 (2.8%)	0
Frontal	1 (2.8%)	3 (8.3%)
Unilateral	3 (8.3%)	3 (8.3%)
Total	29 (80.6 %)	25 (69.4%)
